# Cloning and expression of *Aspergillus flavus* urate oxidase in *Pichia pastoris*

**DOI:** 10.1186/2193-1801-3-395

**Published:** 2014-07-30

**Authors:** Ramin Fazel, Najmeh Zarei, Nasser Ghaemi, Mohammad Mehdi Namvaran, Somayeh Enayati, Esmat Mirabzadeh Ardakani, Mohammad Azizi, Vahid Khalaj

**Affiliations:** Department of Biotechnology, Faculty of Science, University of Tehran, 14174 Tehran, Iran; Medical Biotechnology department, Fungal Biotechnology group, Biotechnology Research Center, Pasteur Institute of Iran, Tehran, Iran; Pharmaceurical Biotechnology department, Shahid Beheshti University of Medical Sciences, Tehran, Iran

**Keywords:** Urate oxidase, Pichia, Rasburicase

## Abstract

Urate oxidase is an important enzyme with therapeutic and diagnostic applications. Rasburicase is a recombinant urate oxidase enzyme approved by FDA to use in the treatment of hyperuricemia conditions. Various hosts such as *Saccharomyces cerevisiae*, *Hansenula polymorpha* and *Escherichia coli* have been used to express the enzyme. Today, *Pichia pastoris* is considered as an important host for heterologous protein expression since it has beneficial characteristics such as strong promoters, simple scale up, post translational modifications, high cell density cultivation and simple genetic manipulation. In this study, *Aspergillus flavus* urate oxidase gene was cloned in pPICZαA expression vector and expressed in *P. pastoris*. The recombinant urate oxidase was expressed in secretory form and was confirmed through RT-PCR, SDS-PAGE analysis and western blotting. The enzyme activity was determined using a colorimetric assay. A production yield of 0.43 U/ml of culture supernatant was obtained.

**Electronic supplementary material:**

The online version of this article (doi:10.1186/2193-1801-3-395) contains supplementary material, which is available to authorized users.

## Background

Urate oxidase (EC 1.7.3.3) plays an important role in purine degradation pathway and catalyzes uric acid oxidation into allantoin, H_2_O_2_ and CO_2_ in the presence of oxygen (Collings et al. 2010). The *Aspergillus flavus* urate oxidase (135 kDa) contains four identical subunits, in which each subunit is associated with one active site. Urate oxidase is a non-glycosylated enzyme having no intra- or inter-disulfide bonds with a blocking acetyl group located at the N-terminal (Legoux et al. 1992).

Urate oxidase is not expressed in human and other higher primates. Although active urate oxidase is detected in some kinds of Old World monkeys, but it is much less active in comparison to urate oxidase in mice and rabbits (Alvarez-Lario and Macarron-Vicente 2010). Although Uric acid is considered to donate more than half the antioxidant capacity of blood resulting in diminution of age-specific cancer and enhancement of life expectancy, but hyperuricemia due to tumor lysis syndrome, excessive dietary purine intake and genetic basis leads to increased risk of hypertension, coronary heart disease, renal dysfunction and gout (Garay et al. 2012).

Since urate oxidase converts uric acid to a more soluble compound, allantoin, it promotes allantoin excretion and prevents uric acid accumulation. Therapeutic effects of urate oxidase by intravenous administration was reported by London and Hudson in 1957 and Rasburicase, a recombinant urate oxidase, was approved after 44 years for treating severe hyperuricemia in patients receiving chemotherapy in Europe and US (Kennedy and Ajiboye 2010).

The urate oxidase of various fungal and non-fungal organisms have been cloned and expressed in different prokaryotic and eukaryotic systems including *E. coli*, *Saccharomyces cerevisiae* and *Hansenula polymorpha*. Rasburicase (Fasturtec) is a recombinant *Aspergillus flavus* urate oxidase, consisting of 301 amino acids, expressed in *S. cerevisiae.* The capability of rasburicase in the reduction of blood uric acid levels and prevention of urate crystals formation in patients is asserted by some surveys (Bosly et al. 2003; Cammalleri and Malaguarnera 2007; Kennedy and Ajiboye 2010).

*Pichia pastoris* is known as a high expressing host (Hsu et al. 2009). Due to the strong methanol-induced alcohol oxidase1 (AOX1) promoter, the small amounts of extra cellular proteins and proteases, the formation of high-copy number integrants and the possibility to select high-copy number colonies by Zeocin™ increasing concentrations, this organism is considered as a highly competitive host for the production of urate oxidase (Arakawa et al. 2006; Daly and Hearn 2005). The first drug produced in *P. pastoris* (Kalbitor) was approved by FDA in 2009 and some other pharmaceuticals including human recombinant serum albumin, hepatitis B vaccine, Botulinum vaccine, Interferon alpha and recombinant human insulin are commercialized (http://pichia.com/science-center/commercialized-products/).

In this study, the *A. flavus* urate oxidase gene was codon optimized according to the codon usage of *P. pastoris* and synthesized commercially. The synthetic gene was cloned into a *Pichia* expression vector containing yeast alpha mating factor. This secretory construct was tagged with 6xHis epitope to facilitate purification steps. To our knowledge, this is the first report on successful expression of heterologous urate oxidase in *P. pastoris*.

## Results

### Preparation of urate oxidase expression cassette

**Figure 1:**
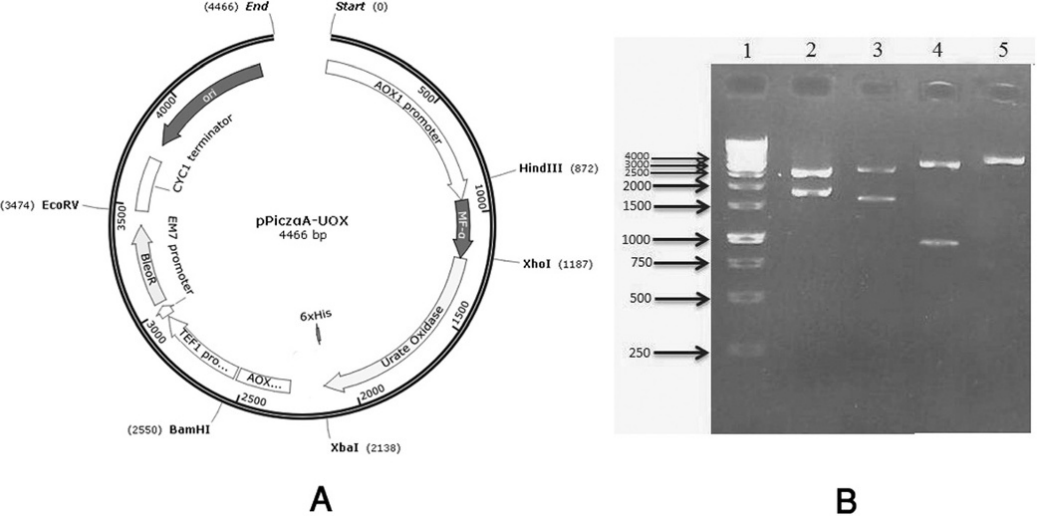
**Restriction analysis of pPICZαA-UOX construct. A**: Schematic representation of pPICZαA-UOX construct map. **B**: Restriction mapping of pPICZαA-UOX plasmid. Lane 1: Size marker. Lane 2: Fragments created by *EcoRV/HindIII* treatment. Two bands of 2602 and 1864 bp are seen. Lane 3: Fragments created by *BamHI*/*HindIII* digestion of the construct with size of 2788 and 1678 bp. Lane 4: The restriction fragments created by *XbaI*/*XhoI* digestion. Two fragments of 3515 and 951 bp are present. Lane 5: *XbaI* linearized construct with a size of 4466.

The expression construct was prepared in order to provide a high level expression of urate oxidase in *P. pastoris*. The codon optimized *A. flavus* urate oxidase gene sequence was synthesized commercially. The sequence alignment of the *A. flavus* urate oxidase and its optimized form is shown in Additional file 1. The synthesized gene segment with the size of 956 bp was cut from pGH-UOX plasmid and successfully cloned into *Xho*I*/Xba*I linearized pPICZαA. Restriction analysis (Figure 1) and DNA sequencing (data not shown) of pPICZαA-UOX plasmid confirmed the cloning procedures and correct orientation of insert inside the vector.

### Transformation of *P. pastoris*and screening of multi copy transformants

*P. pastoris* GS115 strain was transformed with pPICZαA-UOX plasmid using electroporation method and transformants were grown on media containing 200 μg/ml of Zeocin™. In an attempt to further enhancement of protein production levels, we isolated multicopy integrants using the Zeocin screening procedure. The result of PCR on genomic DNA extracted from well grown colonies on Zeocin concentration of 1600 μg/ml showed that pPICZαA-UOX expression cassette is inserted successfully into the GS115 genome. The integration of expression cassette in the genome was confirmed again by PCR using 5′ and 3′ AOX primers (Additional file 2A).

### Expression analysis of urate oxidase

**Figure 2:**
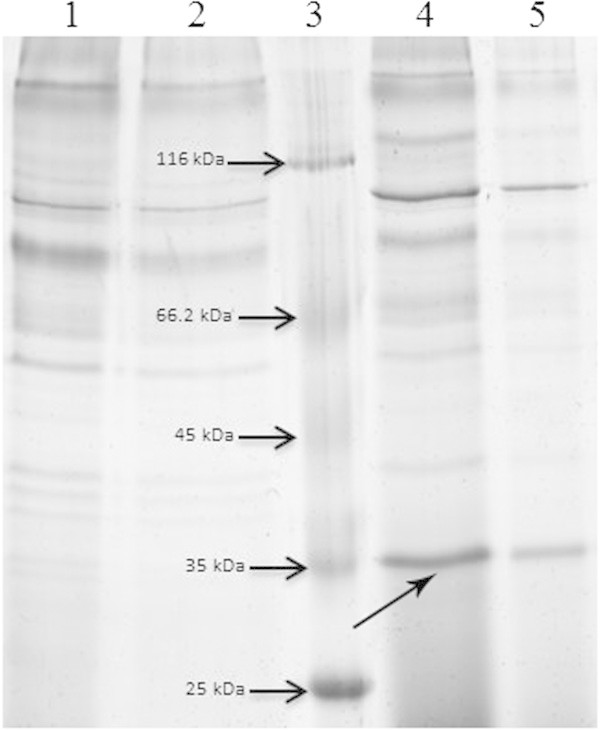
**SDS-PAGE analysis of concentrated supernatants from positive transformant and the control.** Lane 1: Concentrated supernatant of empty vector transformant as negative control. Lane 2: Non-concentrated supernatant of empty vector transformant as negative control. Lane 3: Protein marker. Lane 4: Concentrated supernatant of GS115 positive transformant culture after 4 days of induction. Lane 5: Non-concentrated supernatant of GS115 positive transformant culture after 4 days of induction. Black arrow shows the expressed urate oxidase.

Expression of recombinant urate oxidase was induced by methanol as described in Methods. RT-PCR analysis of methanol induced transformant confirmed the expression of urate oxidase at mRNA level (Additional file 2B).In SDS-PAGE, the expressed band of urate oxidase was clearly detected (Figure 2). Lanes 4 and 5 in Figure 2 show the expressed band of urate oxidase in both concentrated and native supernatants of GS115 positive transformant culture media after 4 days of induction. Lanes 1and 2 are the same as 4&5 but related to the empty vector transformant.

**Figure 3:**
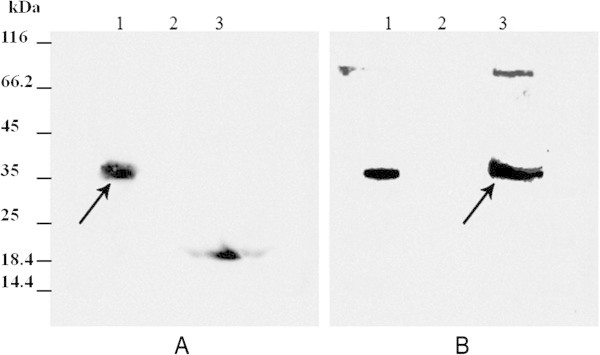
**Western blot analysis on supernatant of GS115 transformant culture media. A**: Western blotting using anti-His tag antibody. Lane 1: Concentrated supernatant of GS115 positive transformant culture media after 4 days of induction. Lane 2: Concentrated supernatant of empty vector transformant. Lane 3: 18 kDa his-tagged protein as a positive control. **B**: Western blotting using anti-rasburacase antibody. Lane 1: positive control (commercial rasburicase), Lane 2: Negative control (Concentrated supernatant of empty vector transformant) and Lane3 Concentrated supernatant of GS115 positive transformant. Black arrows show the expressed urate oxidase.

Densitometry analysis on SDS-PAGE gels demonstrated that the recombinant urate oxidase represents ~24% of total protein in supernatant. This was equal to 37 mg/L of supernatant (See Additional file 3).In western blot analysis of cell-free supernatant using anti-His antibody, a positive band of ~35 kD was detected. Lane 1 in Figure 3A shows the positive band in GS115 transformant culture supernatant and lanes 2 and 3 indicate negative and positive controls, respectively. Figure 3B shows the result of western blot analysis using anti-rasburicase polyclonal antibody. Lane 1 and 3 represent the positive control (commercial rasburicase) and the expressed uricase, respectively.

The His-tagged urate oxidase was purified and used in activity assays (see Additional file 4).

### Urate oxidase activity

The enzyme activity in concentrated supernatants or purified fractions was determined by a spectrophotometric assay as described in Methods. The urate oxidase activity in cell free supernatant was calculated as 0.43 U/ml of 96 h cultures. The activity of purified enzyme was calculated as 11.6U/mg.

## Discussion

High cost is the major disadvantage of treatment by a variety of recombinant pharmaceutics including urate oxidase. Hence, the development of more efficient expression systems to produce large amounts of recombinant proteins very economically is under focus (Cheng Hou et al. 2011).

Eukaryotic cells such as *S. cerevisiae*, *H. polymorpha* and *P. pastoris* are remarkable hosts for the large scale expression of recombinant products, and the superiority of *P. pastoris* in the quality and quantity of expressed proteins has been emphasized in various studies (Cereghino and Cregg 2006). As an example, when the horseradish peroxidase encoding gene expressed in *saccharomyces cerevisiae* was transferred into *P. pastoris*, the expressed enzyme activity increased 5 to 6 times (Morawski et al. 2000). In a review by Valero, a prokaryotic system like *Escherichia coli* and some eukaryotic systems such as *S. cerevisiae* and *P. pastoris* are analyzed and compared in terms of productivity and downstream processing facilities for lipase expression (Valero 2012). According to this review, the *P. pastoris* expression system has been shown to be the most promising system.

In the majority of published reports, the *E. coli* expression system has been used for the recombinant production of urate oxidase (Legoux et al. 1992); (Nakagawa et al. 1995) (Li et al. 2006). The maximum expression obtained in *E. coli* has been 238 mg/L which is obviously less than the expression quantities in yeasts (Li et al. 2006). Usually in prokaryotic expression systems initiating methionine remains in enzyme structure and affects both the constancy and activity of the enzyme and also the therapeutical applications will fail (Valero 2012).

In registered patent of Sanofi-Aventis Company, the amount of urate oxidase expression in *S. cerevisiae* cell lysate is 0.255 mg/ml which is not high enough (Caput et al. 1995). Furthermore, as a result of high level of impurities (3 mg/ml) in the mixture of cell lysate, costly purification steps would be essential.

According to Chen et al., the only appropriate amount of urate oxidase expression in yeast has been achieved in *H. polymorpha* (Chen et al. 2008). In their report, the *Candida utilis* urate oxidase was expressed by *H. polymorpha* and α-MF signal sequence of *S. cerevisiae* was used to secret the protein. The preliminary expression level in shake flask was 0.3 U/ml of culture medium. Further optimization of the fermentation process resulted in increased levels up to 174 times, giving an extracellular expression level of 2.1 g/L and an intracellular expression of 2.4 g/L. However, the higher level of intracellular protein compared to the extracellular protein implicates a problem during the secretion of the recombinant protein into the culture medium. In a recent study by Dmytruk and colleagues, the recombinant urate oxidase activity expressed in *H. polymorpha* showed lower activity compared to the commercially available enzyme (Dmytruk et al. 2011).

In our study, the amount of expressed urate oxidase in *P. pastoris* is about 24.2% of total supernatant proteins, while the produced urate oxidase in *S. cerevisiae* is 8.5% of the total lysate proteins. Although the expression level of 0.4 U/ml in shaking flask is promising, however more optimization is required. The activity of purified recombinant urate oxidase was 11.6 U/mg which is less than commercially available enzyme (18.2 U/mg). This can be due to cultivation techniques and purification conditions which are reported to be important in productivity, stability and activity of the recombinant proteins (Macauley-Patrick et al. 2005). The higher expression and activity of urate oxidase by selecting the more efficient colonies and the optimization of the fermentation and purification conditions can be expected.

## Conclusion

The presented data show the ability of *P. Pastoris* in the expression of active recombinant urate oxidase (UOX). The Recombinant production of the enzyme in this methylotrophic yeast may be translated into the development of cost-effective manufacturing processes for the production of UOX biosimilars.

## Methods

### Strains, reagents and culture media

*Pichia pastoris* (Invitrogen) strain GS115 as the expression host and pPICZαA (Invitrogen) as the expression vector were used for heterologous protein expression. *Escherichia coli* strain Top10 (Invitrogen) cells were used in DNA recombinant procedures. Buffered complex medium, containing glycerol (BMGY; Invitrogen) was used for growing the cells before induction and buffered complex medium containing methanol (BMMY; Invitrogen) was used as induction medium. Restriction enzymes were purchased from Fermentase. Anti-His antibody for detection of recombinant histidine-tagged urate oxidase was purchased from Roche Applied Science. Other reagents were obtained from standard commercial sources.

### Construction of expression vector

The *A. flavus* urate oxidase gene sequence was obtained from database and codon optimized according to the *P. pastoris* codon usage*.* To express urate oxidase protein with a native N terminus, the *Xho*I site at base pairs 1184–1189 of the expression vector pPICZαA (Invitrogen easy select *Pichia* Expression kit) was used to clone the gene flush with the Kex2 cleavage site. So this sequence (CTCGAGAAAAGAGAGGCTGAAGCT) was included in upstream of the urate oxidase gene sequence and polyhistidine (6X) tag sequence was added to the downstream of urate oxidase gene before stop codon. The modified synthetic gene (GeneRayBiotech, Shanghai) was supplied as a circularized plasmid, pGH–UOX, containing all the necessary restriction sites for directional cloning into pPICZαA. The urate oxidase gene was cut out from pGH-UOX using *Xho*I/*Xba*I double digestion and subsequently cloned into *Xho*I/*Xba*I site of pPICZαA. The final construct was called pPICZαA-UOX and confirmed by restriction analysis and sequencing.

### Transformation of GS115 cells

Ten micrograms of *SacI* linearized pPICZαA-UOX plasmid was used in transformation of GS115 cells using an electroporation method provided by the manufacturer (Invitrogen easy select Pichia Expression Kit). The cells were washed in two successive steps with sterilized water and 1 M sorbitol based on manufacturer protocol. Following the electroporation step, the transformants were selected on YPDS agar medium containing Zeocin (200 μg/ml). Increasing concentrations of Zeocin™ were used to isolate multi-copy transformants. This screening procedure was carried out by re-plating the grown colonies on various increasing concentrations of Zeocin from 400 μg/ml to 1600 μg/ml. Genomic DNA of transformants was extracted and the presence of expression cassette was confirmed by urate oxidase specific primers: UOX_f (5′- TCCGCAGTTAAAGCTGC-3′) and UOX_r (5′-CAATTTAGACTTCAGAGAGG-3′). The second confirmatory PCR was carried out with primers spanning the AOX1 elements (AOX_f: 5′-GACTGGTTCCAATTGACAAGC-3′ and AOX_r: 5′-GAAATGGCATTCTGACATCC-3′).

### The expression of recombinant urate oxidase in GS115 strain

A single colony of GS115 transformant was grown in 50 ml of BMGY at 30°C in a shaking incubator (250 rpm). The cells (OD_600_ = 4) were harvested and resuspended in 200 ml of BMMY in a 1 L triple side baffled flask (OD_600_ = 1.0) to induce expression at 30°C. Absolute methanol was added to a final concentration of 0.5% every 24 h to maintain the induction.

### RNA isolation and cDNA synthesis

Two single colonies of GS115 transformed with pPICZαA and pPICZαA-UOX vectors were grown under methanol induction as described. Total RNA was isolated using the RNeasy Plus mini kit (Qiagen) and cDNA was synthesized from RNA using RevertAid First Strand cDNA Synthesis Kit (Fermentase) according to the manufacturer’s instructions. PCR reaction using UOX primers was done on cDNA of an empty vector transformed GS115 strain as negative control and pPICZαA-UOX transformed strain to detect any urate oxidase transcripts.

### SDS-PAGE, western blotting and purification

Supernatant of BMMY expression culture medium was harvested and then was concentrated ten to hundred fold using an Amicon stirred ultrafiltration cell 8400 equipped with a 10 KDa cut off filter (Millipore, USA). The electrophoretic separation of proteins was carried out in a 12% polyacrylamide gel and western blotting was performed according to the standard methods. The concentrated supernatant of GS115 transformed with empty vector (pPICZαA) was used as the negative control in both tests. A rabbit polyclonal antibody against commercial urate oxidase was prepared using the standard procedures (Cooper and Paterson 2008). This antibody was used in confirmatory western blot analysis. Anti-His antibody was also used in western blot analyses to confirm His-tagged urate oxidase expression. An 18 kDa His-tagged protein was used as a positive control. The recombinant His-tagged urate oxidase was purified from the supernatants by affinity chromatography using an Ni-NTA purification system (Quiagen, USA) Equilibration of Ni-NTA column, washing of weakly bound proteins and elution of the purified protein were performed using 10 mM, 20 mM and 300 mM imidazole buffer, respectively. The Bradford assay was used in protein quantifications. Densitometry on SDS-PAGE gels was done using Quantity One software to measure the expression level of secreted urate oxidase.

### Urate oxidase assay

The urate oxidase activity was determined using a modified urate oxidase/phenol/4-aminoantipyrine colorimetric assay (Klose et al. 1987)). Briefly, uric acid can be specifically converted to allantoin and hydrogen peroxide by urate oxidase. Subsequently, the resulting hydrogen peroxidase reacts with 4-aminoantipyrine in the presence of peroxidase to generate a pink color product with an optimum absorption in 505 nm. The intensity of the produced color is directly proportional to urate oxidase activity.

300 μl of the concentrated cell free supernatant or purified urate oxidase fraction was added to a reaction mixture containing 100 μl uric acid solution (6 mM in borate buffer, pH 8.6), 50 μl horseradish peroxidase (15 U/ml in phosphate buffer), 1.5 μl phenol and 150 μl 4-aminoantipyrine (30 mM). The reaction was incubated at 37°C for 30 minutes.

The increasing concentrations of commercial urate oxidase (Fasturtec: Sanofi Aventis, 2.7 U/ml) were added to assay mixtures as above and the corresponding optical densities were measured to construct the standard curve. The enzyme activity of samples was calculated using this standard curve (Additional file 5).

## Electronic supplementary material

Additional file 1: **A Sequence alignment of** ***A. flavus*** **urate oxidase and its optimized form.** The asterisks indicate identical bases. (PDF 480 KB)

Additional file 2: **PCR and RT-PCR analysis of UOX transformant. A.** Genomic amplification of urate oxidase expression unit using AOX specific primers. Lane 1: PCR product amplified from a pPICZαA-UOX positive Pichia transformant (~1.5 kb), Lane 2: Positive control (pPICZαA-UOX plasmid), Lane 3: Negative control (genomic DNA from an empty vector transformant), Lane 4: Size marker. **B**: RT-PCR analysis of pPICZαA-UOX positive transformant using UOX primers. Lane 1: An approximately 750 bp amplified fragment from a positive Pichia transformant, Lane 2: No amplification in negative control (empty vector transformant), Lane 3: Positive control (pPICZαA-UOX plasmid), Lane 4 : The same as 1 but with 1:10 dilution of cDNA, Lane 5: PCR result on RNA (No amplification) and Lane 6: Size marker. (JPEG 158 KB)

Additional file 3: **Densitometry analysis of proteins on SDS-PAGE of UOX positive transformant culture supernatant.** Band 18 (expressed urate oxidase) constitutes ~24% of total proteins. (JPEG 380 KB)

Additional file 4: **SDS –PAGE analysis of Purified His-tagged urate oxidase.** Gel was stained with coomassie blue. Lane1: Positive control, Lane2: Size marker and Lane 3: purified protein. (JPEG 42 KB)

Additional file 5: **A standard curve used in urate oxidase activity assay.** As described in methods, the different volumes of standard stock (Rasburicase, 0.1 mg/ml, 2.7 U/ml) were added to the reaction mixtures and optical densities were measured at 505 nm. (JPEG 36 KB)
