# Supplementary material for: Cloning and expression of Aspergillus flavus urate oxidase in Pichia pastoris
Source: Springerplus. 2014 Jul 30;3:395. doi: 10.1186/2193-1801-3-395 (PMC4124111; doi:10.1186/2193-1801-3-395)
Supplement: Supplementary file 1 — Additional file 1: A Sequence alignment of A. flavus urate oxidase and its optimized form. The asterisks indicate identical bases. (PDF 480 KB) [file 40064_2014_1090_MOESM1_ESM.pdf]

```
CLUSTAL O(1.2.1) multiple sequence alignment


original        ATGTCCGCAGTAAAAGCAGCCCGCTACGGCAAGGACAATGTCCGCGTCTACAAGGTTCAC    60
optimized       ATGTCTGCCGTCAAAGCTGCCAGATACGGTAAAGACAATGTCAGAGTTTACAAAGTCCAT    60
                ***** ** ** ***** *** * ***** ** ********* * ** ***** ** **

original        AAGGACGAGAAGACCGGTGTCCAGACGGTGTACGAGATGACCGTCTGTGTGCTTCTGGAG    120
optimized       AAGGACGAAAAGACTGGTGTCCAGACCGTTTACGAAATGACTGTCTGCGTCTTGTTGGAG    120
                ******** ***** *********** ** ***** ***** ***** **  *  *****

original        GGTGAGATTGAGACCTCTTACACCAAGGCCGACAACAGCGTCATTGTCGCAACCGACTCC    180
optimized       GGTGAAATCGAAACTTCTTACACAAAGGCCGATAATTCTGTCATCGTTGCCACTGACTCT    180
                ***** ** ** ** ******** ******** **    ***** ** ** ** *****

original        ATTAAGAACACCATTTACATCACCGCCAAGCAGAACCCCGTTACTCCTCCCGAGCTGTTC    240
optimized       ATCAAGAACACCATCTACATCACCGCTAAACAGAACCCAGTTACCCCACCTGAGTTGTTC    240
                ** *********** *********** ** ******** ***** ** ** *** *****

original        GGCTCCATCCTGGGCACACACTTCATTGAGAAGTACAACCACATCCATGCCGCTCACGTC    300
optimized       GGTTCTATCTTGGGTACTCACTTCATTGAGAAATACAACCACATCCATGCCGCCCACGTT    300
                ** ** *** **** ** ************** ******************** *****

original        AACATTGTCTGCCACCGCTGGACCCGGATGGACATTGACGGCAAGCCACACCCTCACTCC    360
optimized       AATATCGTCTGTCACAGATGGACAAGAATGGACATCGACGGAAAGCCTCATCCTCACTCT    360
                ** ** ***** *** * *****  * ******** ***** ***** ** ********

original        TTCATCCGCGACAGCGAGGAGAAGCGGAATGTGCAGGTGGACGTGGTCGAGGGCAAGGGC    420
optimized       TTCATCAGAGACTCTGAGGAGAAAAGAAATGTCCAAGTCGACGTTGTCGAGGGTAAGGGA    420
                ****** * ***   ********  * ***** ** ** ***** ******** *****

original        ATCGATATCAAGTCGTCTCTGTCCGGCCTGACCGTGCTGAAGAGCACCAACTCGCAGTTC    480
optimized       ATTGACATTAAGTCTTCTTTGTCTGGATTGACAGTCTTGAAGTCTACAAATTCTCAGTTT    480
                ** ** ** ***** *** **** **  **** **  *****   ** ** ** *****

original        TGGGGCTTCCTGCGTGACGAGTACACCACACTTAAGGAGACCTGGGACCGTATCCTGAGC    540
optimized       TGGGGTTTCTTGAGAGATGAATACACAACCTTGAAGGAAACCTGGGATAGAATCTTGTCT    540
                ***** *** ** * ** ** ***** **  * ***** ********  * *** **

original        ACCGACGTCGATGCCACTTGGCAGTGGAAGAATTTCAGTGGACTCCAGGAGGTCCGCTCG    600
optimized       ACCGATGTTGACGCCACTTGGCAATGGAAGAACTTTTCTGGTTTGCAGGAGGTCAGATCT    600
                ***** ** ** *********** ******** **   ***  * ********* * **

original        CACGTGCCTAAGTTCGATGCTACCTGGGCCACTGCTCGCGAGGTCACTCTGAAGACTTTT    660
optimized       CACGTTCCTAAGTTCGATGCCACATGGGCAACCGCCAGAGAGGTCACTTTGAAAACTTTC    660
                ***** ************** ** ***** ** **  * ********* **** *****

original        GCTGAAGATAACAGTGCCAGCGTGCAGGCCACTATGTACAAGATGGCAGAGCAAATCCTG    720
optimized       GCCGAAGATAACTCTGCCTCTGTTCAAGCAACCATGTACAAAATGGCCGAGCAGATCTTG    720
                ** *********  ****   ** ** ** ** ******** ***** ***** *** **

original        GCGCGCCAGCAGCTGATCGAGACTGTCGAGTACTCGTTGCCTAACAAGCACTATTTCGAA    780
optimized       GCTAGACAGCAGTTGATTGAAACCGTCGAATACTCTTTGCCAAACAAACACTACTTCGAG    780
                **  * ****** **** ** ** ***** ***** ***** ***** ***** *****

original        ATCGACCTGAGCTGGCACAAGGGCCTCCAAAACACCGGCAAGAACGCCGAGGTCTTCGCT    840
```

```
optimized       ATTGACTTGTCTTGGCACAAGGGTTTGCAGAACACTGGAAAGAATGCAGAGGTTTTCGCT    840
                ** *** **   ***********  * ** ***** ** ***** ** ***** ******

original        CCTCAGTCGGACCCCAACGGTCTGATCAAGTGTACCGTCGGCCGGTCCTCTCTGAAGTCT    900
optimized       CCACAGTCTGACCCAAACGGTTTGATTAAGTGCACCGTTGGTAGATCTTCTTTGAAGTCT    900
                ** ***** ***** ****** **** ***** ***** **  * ** *** ********

original        AAATTGT  907
optimized       AAGTTGC  907
                ** ***
```
