# Supplementary figures and images for: Cloning and expression of Aspergillus flavus urate oxidase in Pichia pastoris
Source: Springerplus. 2014 Jul 30;3:395. doi: 10.1186/2193-1801-3-395 (PMC4124111; doi:10.1186/2193-1801-3-395)

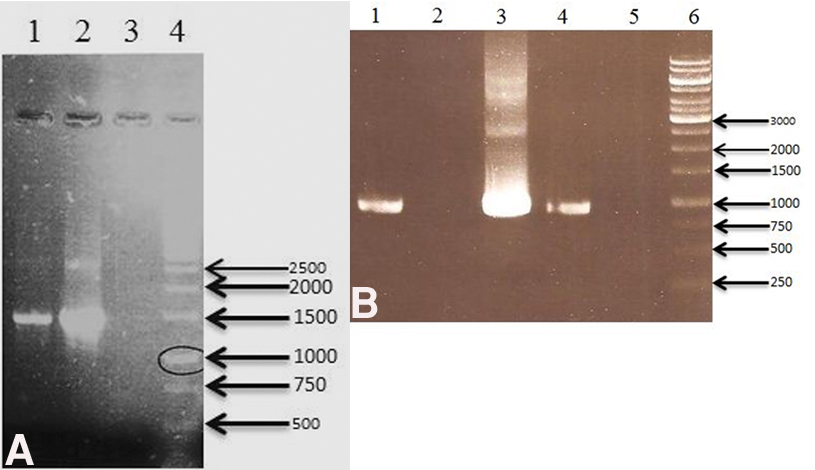

Supplement: Supplementary file 2 — Additional file 2: PCR and RT-PCR analysis of UOX transformant. A. Genomic amplification of urate oxidase expression unit using AOX specific primers. Lane 1: PCR product amplified from a pPICZαA-UOX positive Pichia transformant (~1.5 kb), Lane 2: Positive control (pPICZαA-UOX plasmid), Lane 3: Negative control (genomic DNA from an empty vector transformant), Lane 4: Size marker. B: RT-PCR analysis of pPICZαA-UOX positive transformant using UOX primers. Lane 1: An approximately 750 bp amplified fragment from a positive Pichia transformant, Lane 2: No amplification in negative control (empty vector transformant), Lane 3: Positive control (pPICZαA-UOX plasmid), Lane 4 : The same as 1 but with 1:10 dilution of cDNA, Lane 5: PCR result on RNA (No amplification) and Lane 6: Size marker. (JPEG 158 KB) [file 40064_2014_1090_MOESM2_ESM.jpeg]

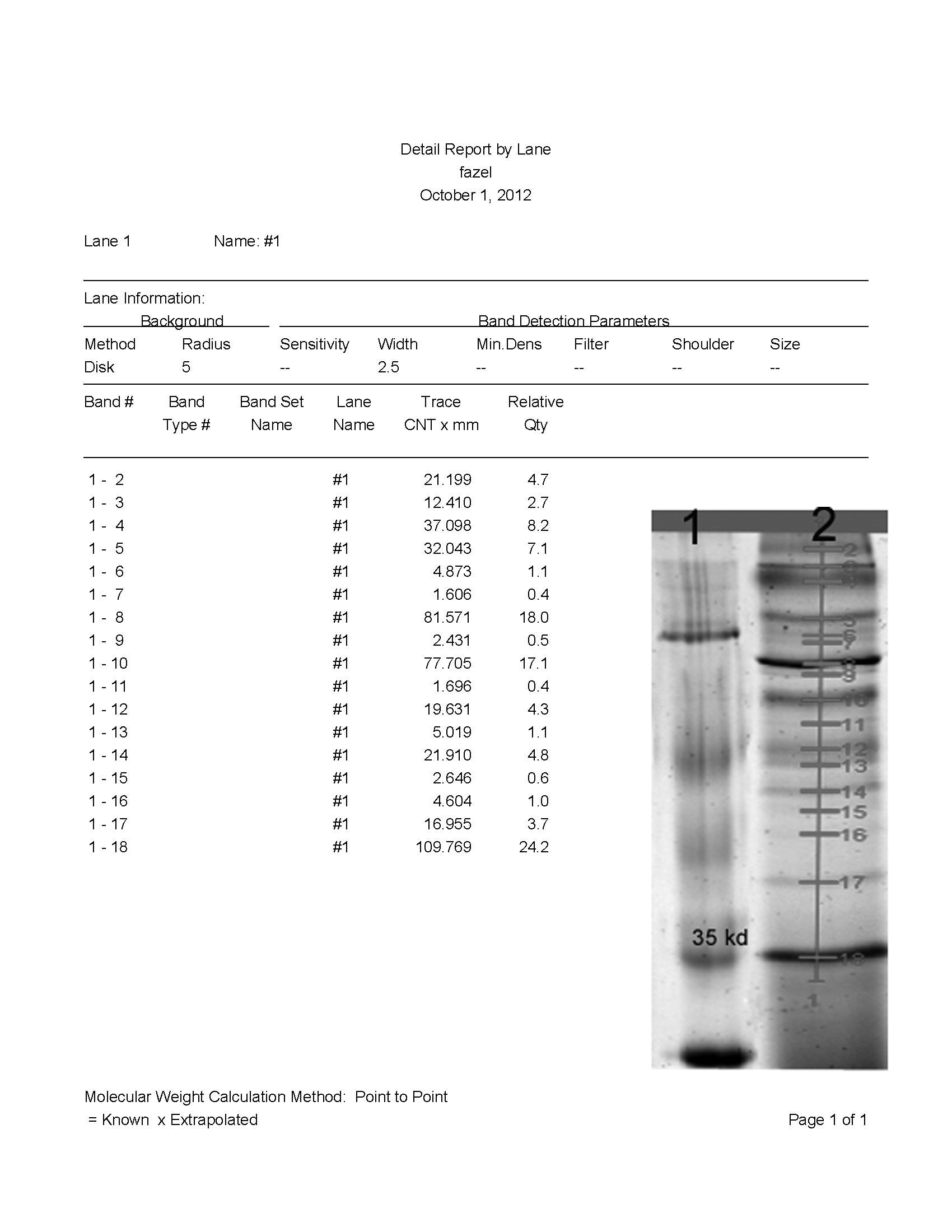

Supplement: Supplementary file 3 — Additional file 3: Densitometry analysis of proteins on SDS-PAGE of UOX positive transformant culture supernatant. Band 18 (expressed urate oxidase) constitutes ~24% of total proteins. (JPEG 380 KB) [file 40064_2014_1090_MOESM3_ESM.jpeg]

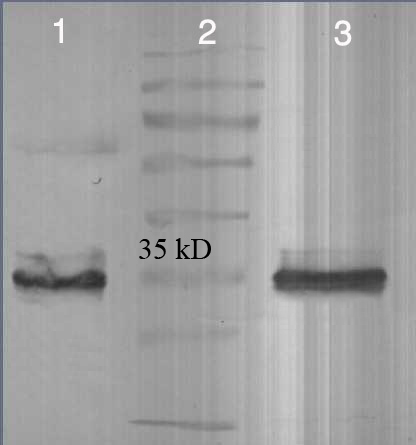

Supplement: Supplementary file 4 — Additional file 4: SDS –PAGE analysis of Purified His-tagged urate oxidase. Gel was stained with coomassie blue. Lane1: Positive control, Lane2: Size marker and Lane 3: purified protein. (JPEG 42 KB) [file 40064_2014_1090_MOESM4_ESM.jpeg]

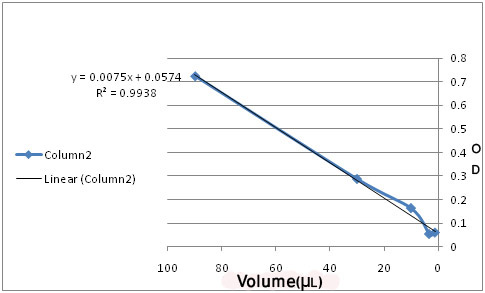

Supplement: Supplementary file 5 — Additional file 5: A standard curve used in urate oxidase activity assay. As described in methods, the different volumes of standard stock (Rasburicase, 0.1 mg/ml, 2.7 U/ml) were added to the reaction mixtures and optical densities were measured at 505 nm. (JPEG 36 KB) [file 40064_2014_1090_MOESM5_ESM.jpeg]
